# Supplementary material for: Understanding the Effects of Lactose Hydrolysis Modeling on the Main Oligosaccharides in Goat Milk Whey Permeate
Source: Molecules. 2019 Sep 10;24(18):3294. doi: 10.3390/molecules24183294 (PMC6767524; doi:10.3390/molecules24183294)
Supplement: Supplementary file 1 [file molecules-24-03294-s001.pdf]

molecules

Supplemental materials

Table S1. Variables and levels evaluated in the experimental design to optimize lactose hydrolysis efficiency by *A. oryzae* β-galactosidase and preservation/formation of oligosaccharides.

Variable	Level				
	−1.41	−1	0	1	+1.41
Temperature (°C), x^1	25.9	30	40	50	54.1
Enzyme (%), x^2	0.18	0.20	0.25	0.30	0.32

[1] Complete 2^2 factorial design parameters, with 2 independent variables in 2 levels, 3 repetitions in the central point, and 4 axial points.

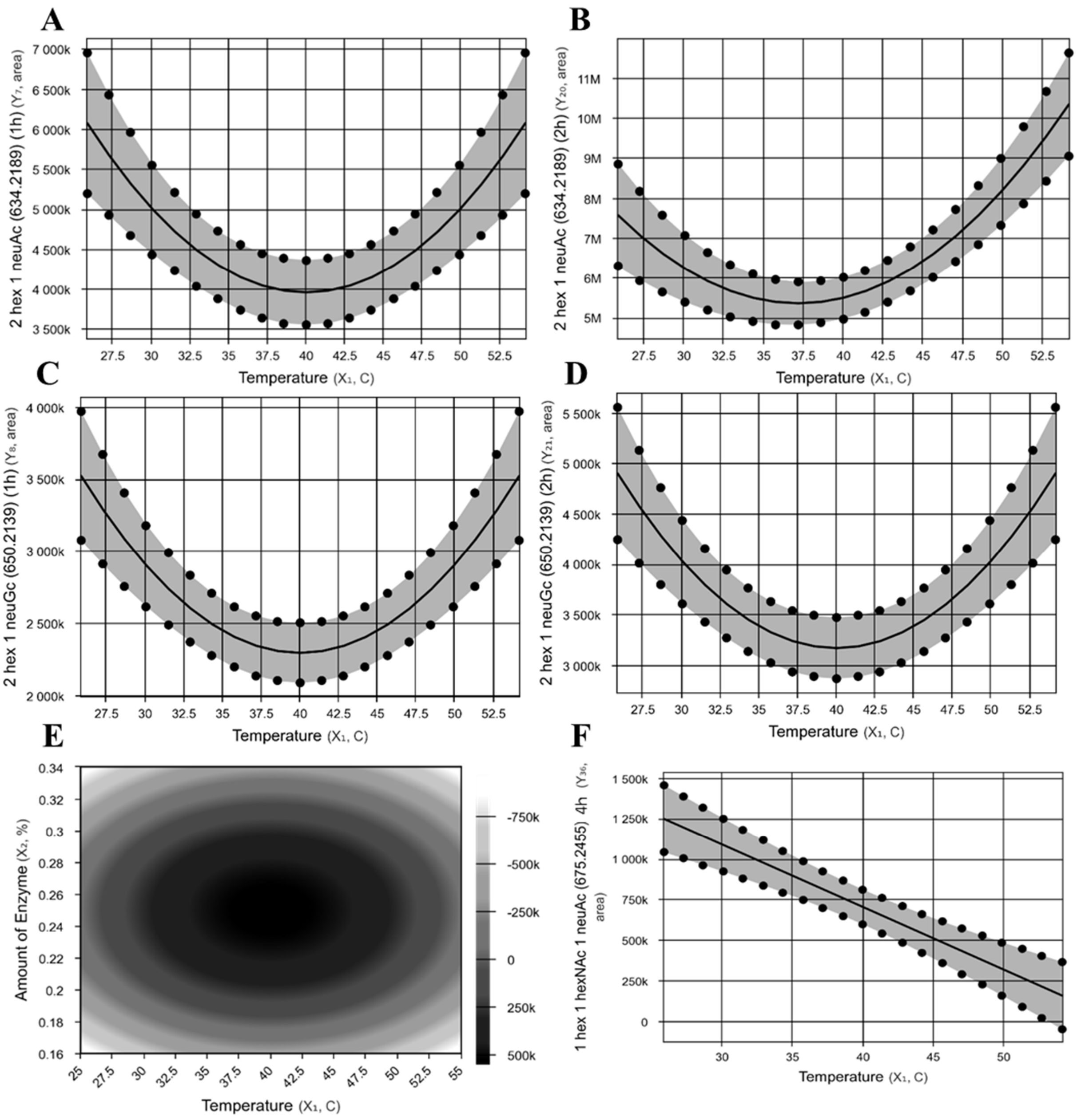

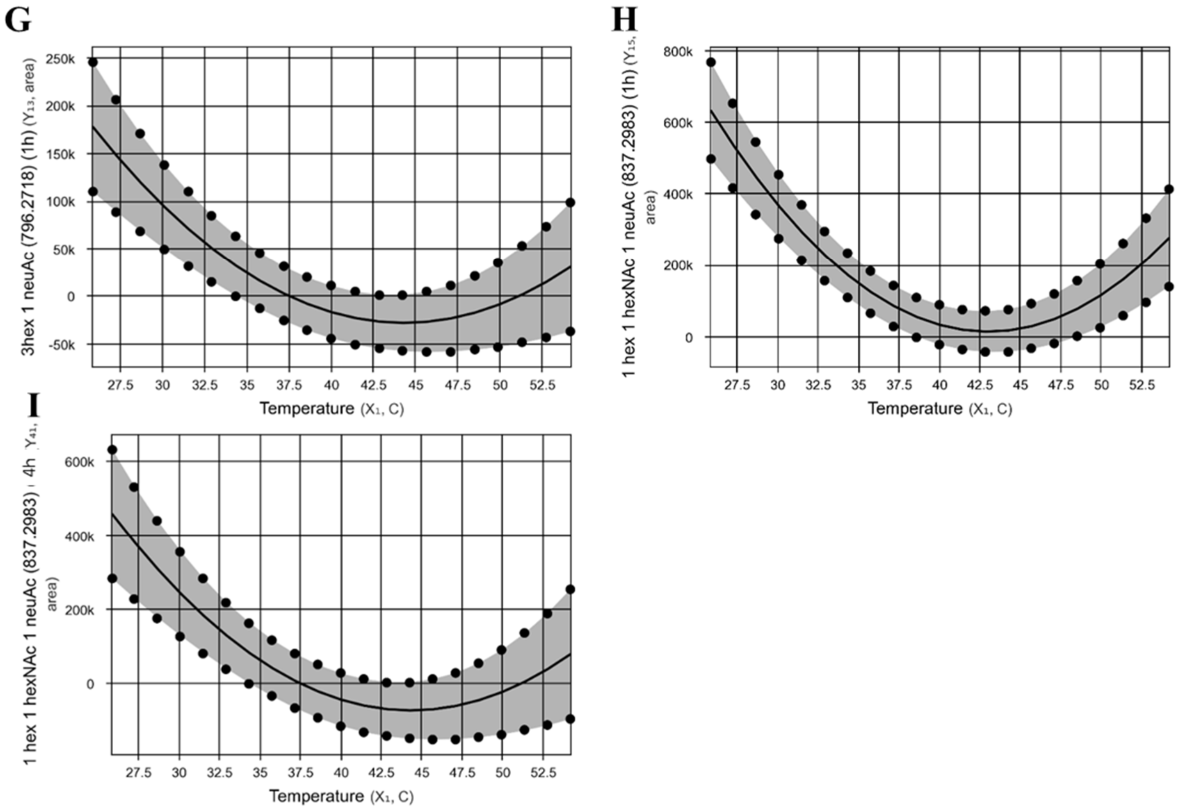

Figure S1. Contour curve for the abundance of the acidic oligosaccharides (**A**), 2 Hex 1 NeuAc (634.2189) (1 h); (**B**), 2 Hex 1 NeuAc (634.2189) (2 h); (**C**), 2 Hex 1 NeuGc (650.2139) (1 h); (**D**), 2 Hex 1 NeuGC (650.2139) (2 h); (**E**), 1 Hex 1 Hex Nac 1 NeuAc (675.2455) (2 h); (**F**), 1 Hex 1 HexNAc 1 NeuAc (675.2455) (4 h); (**G**), 3 Hex 1 NeuAc (796.2718) (1 h); (**H**), 2 Hex 1 HexNAc 1 NeuAc (837.2983) (1 h); (**I**), 2 Hex 1 HexNAc 1 NeuAc (837.2983) (4 h).

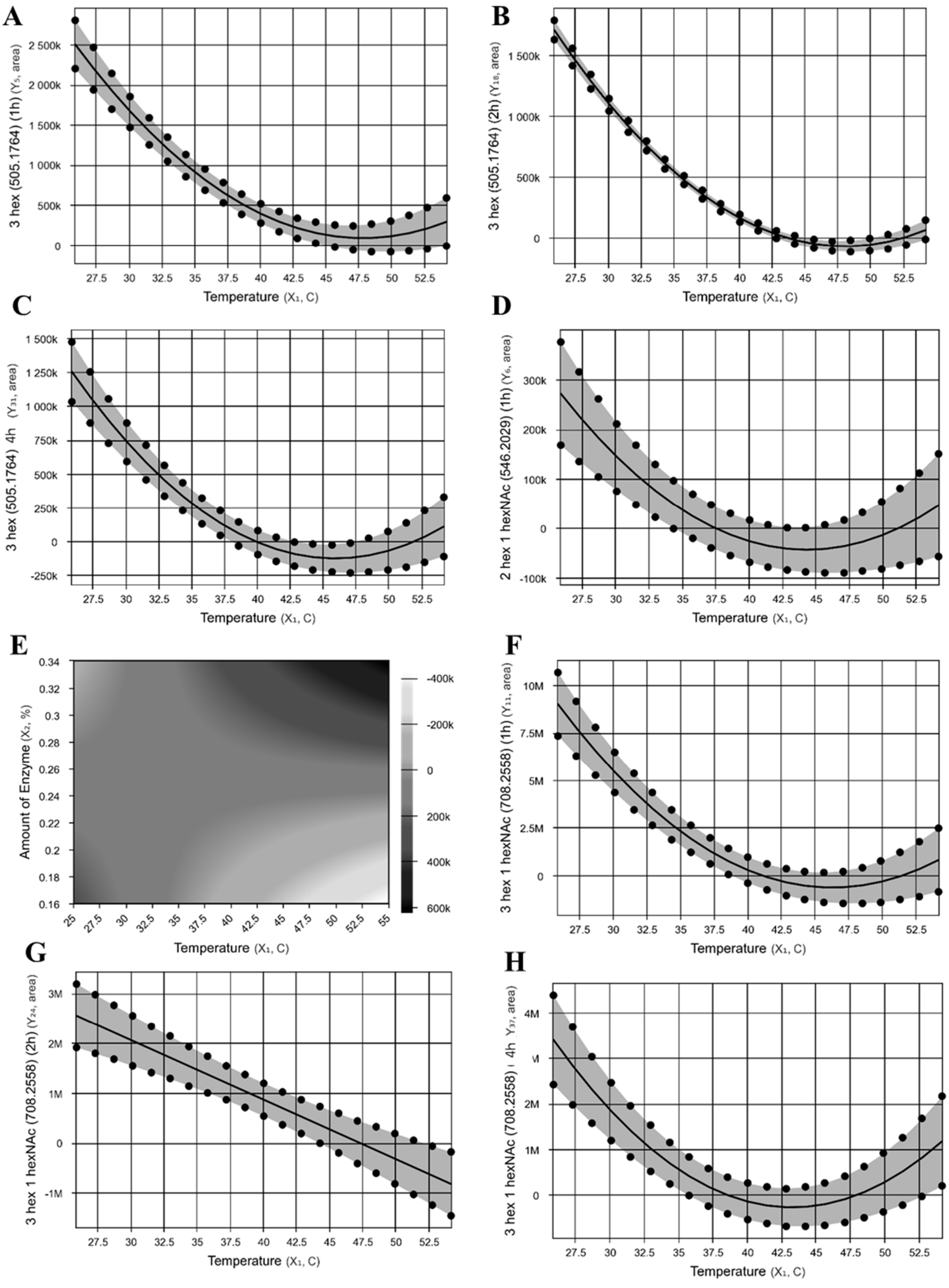

Figure S2. Contour curve for the abundance of the neutral oligosaccharides (**A**), 3 Hex (505.1764) (1 h); (**B**), 3 Hex (505.1764) (2 h); (**C**), 3 Hex (505.1764) (4 h); (**D**), 2 Hex 1 HexNAc (546.2029) (1 h); (**E**), 2 Hex 1 HexNAc (546.2029) (4 h), (**F**), 3 Hex 1 HexNAc (708.2558) (1 h); (**G**), 3 Hex 1 HexNAc (708.2558) (2 h); (**H**), 3 Hex 1 HexNAc (708.2558) (4 h); (**I**), 2 Hex 2 HexNAc (749.2823) (1 h); (**J**), 2 Hex 2 HexNAc (749.2823) (4 h).
